# Psychosocial work environment and health when entering or leaving a managerial position

**DOI:** 10.3233/WOR-210469

**Published:** 2022-10-17

**Authors:** Daniel Lundqvist

**Affiliations:** Unit of Education and Sociology, Department of Behavioural Sciences and Learning, Linköping University, Linköping, Sweden E-mail: daniel.lundqvist@liu.se

**Keywords:** Leaders, job characteristics, well-being, job transitions, longitudinal study

## Abstract

**BACKGROUND:**

Recruiting and retaining managers has become increasingly difficult in recent years, primarily because of a pressured work situation. A better understanding of managers’ work situation is required, and of the support they need.

**OBJECTIVE:**

The purpose of the study is to increase the understanding of managers’ psychosocial work environment and health by investigating individuals as they enter or leave a managerial position.

**METHODS:**

Longitudinal questionnaire data from 1971 individuals distributed across four groups were used: individuals who 1) entered or 2) left a managerial position between measuring points, and those who remained employed as 3) managers or 4) non-managers at both measuring points.

**RESULTS:**

Demands increased between the measuring points for those who entered a managerial position. Their resources and health were, however, rated higher than non-managers already before the transition. Demands decreased for those who left a managerial position, while their resources remained higher than non-managers. Health did not change by changing position.

**CONCLUSION:**

This study contributes to knowledge of what happens when someone enters or leaves a managerial position and increases the understanding of differences between managers and non-managers. Organizations should develop supportive strategies through talent management programs to help build resources in employees and future managers. Support should also aim to reduce the increased level of demands in newly hired managers.

## 1 Introduction

Managers serve an important function in organizations, and their decisions and actions can have far-reaching consequences for the organization and its employees [1, 2]. It is crucial to create favourable conditions to help them perform their work. However, managers have often been found to have a highly stressful work situation [3–7], which is the primary reason why it has become increasingly difficult to recruit managers in Sweden and other parts of Europe [8, 9]. In order to recruit and retain newly hired managers, more knowledge is needed on managers’ work situation and the support they need.

Research on work environment and health shows that resources at work are important in several ways. Resources can be used to manage stressful work situations but can also create learning and development of skills at work [10]. Managers generally experience their work as more demanding and stressful than non-managers, but they also have better health [11–15]. One explanation to why managers have better health is that the managerial position offers access to more resources that promote health. A second opposing explanation is that individuals who already have access to resources and good health are the ones who get hired as managers. In order to help newly hired managers and give them relevant support, a better understanding of what happens when someone enters or leaves a managerial position is needed [16].

The purpose of this study is to increase the understanding of managers’ psychosocial work environment and health by testing the two explanations on individuals entering or leaving a managerial position. The demands-control-support model [10] is used as a theoretical starting point. The model consists of demands that may cause stress and illness, and resources in terms of control and support that can be used to ease the demands at work.

This study contributes to the literature in several ways. It answers the call [16] for more attention to two specific groups – those who enter and those who leave a managerial position. In this way, a better picture can be given of how work conditions and health can be affected when changing position. The study also contributes to a clearer understanding of whether health is enhanced by the position or brought into the position. Knowledge of how different work situations contribute to health can increase the understanding of the differences between managers and non-managers and the different conditions they have in working life [11–15]. This is an important issue as it helps organizations to promote sustainable and long-term employment within the organization and possibly decrease the problems of recruiting managers.

The following section provides a review of the literature on managers’ work situation, and two alternative explanations for understanding managers’ health are described. Hypotheses are then formulated based on these two explanations. The method and the empirical material are described in the subsequent section. The results are thereafter presented, followed by a discussion. The paper ends with study limitations and conclusions.

### 1.1 Literature review

#### 1.1.1 Demands-control-support model and health

The demands-control-support model is one of the most well-researched models to study the psychosocial work environment and health in working life [17–20]. The original model focused on two different aspects of the work environment: level of demands and level of control [21]. Demands is a generic measure of three stressors – time pressure, role conflicts, and workload, while control consists of two resources at work – level of skill and creativity, and level of autonomy in decisions. The model combines high and low demands with high and low control to depict four work situations: active jobs (high demands, high control), low-strain jobs (low demands/high control), passive jobs (low demands, low control), high-strain jobs (high demands/low control). Social support, focusing on the social relationships and climate at work, was added later to the model to form the demands-control-support model [10, 22]. Work situations high in control and support are generally suggested to be beneficial to the employee’s health and development, while work situations low in control and support, especially in combination with high demands, are detrimental to the employee’s health and development. The latter suggestion has received support in several reviews and meta-analyses [17–20].

The demands-control-support model has been used in relation to several different measures of health, such as burnout, cardiovascular disease, and morbidity [17–20]. Health is, however, a complex concept, and there are several competing theories and definitions: health as normal functioning, as ability, or as well-being [23, 24]. The lack of a common definition makes health difficult to operationalize and measure, and various indicators are therefore used in research [25]. Indicators of health can be based on a pathogenic perspective and focus on different types of complaints, ailments, and negative experiences, or they can be based on a salutogenic perspective and focus on energy, satisfaction, and other positive experiences [26, 27].

#### 1.1.2 Research on managers’ work situation and health

Studies focusing on managers’ psychosocial work environment and health show that their work is highly demanding with fragmented, varied and often complex and interdependent work tasks [3–7]. Role conflicts, role ambiguities and personnel issues are, for example, common. Only a few studies have compared managers’ and subordinates’ work environment, and these show that managers perceive higher demands in their work compared to their subordinates [11, 12, 28, 29]. These studies further show that managers perceive higher control, autonomy, influence, and freedom in their work [11, 12, 28–30]. Thus, the managerial role is subject to high demands but at the same time offer the resources to deal with these demands [28, 31–34].

Translated to the demands-control-support model [10], managers’ work is characterized as an active work situation, high in demands, control, and support [28, 31]. Previous research shows that the healthiest work is found in the ideal (low-strain) work situation or the active work situation, i.e., situations high in control and support [17–20]. Managers generally have better health compared to non-managers [11–15, 35], even after considering socio-economic factors, such as education and income. Managers’ better health is usually explained by the managerial position offering access to more resources to handle stressful work situations [11, 29, 31, 35], here referred to as the favourable condition explanation.

A different stand on the issue is to focus on the individuals that are being hired as managers. Showing initiative and drive is regarded as central for a career as a manager [36–38]. Characteristics found in research about organizational talents and individuals with high potential for a managerial position include having high development potential, willingness to learn, and being able to identify learning opportunities [39–43]. Other characteristics involve being energetic, competent, social, creative, flexible, committed, and high performing. However, previous research shows that to be able to learn, to be creative, and to be high performing etc., the work environment and the individual’s own health needs to allow and support those kinds of characteristics. For instance, networking and maintaining social relations are vital for a managerial career [37, 38, 44], but that requires sufficient health and a work environment that makes it possible to network [36, 45, 46]. An individual with high demands and low influence is less able to network and socialize.

It would seem that people looking for a managerial position are likely highly motivated, committed and perceive a work environment that supports their motivation. Similarly, people that do not show that they are motivated and committed are less likely considered for a managerial position by the employer [47, 48]. The differences between managers and non-managers in perceived work environment and health that previous studies have found can thus potentially be explained by who gets hired as managers, here referred to as the selection effect explanation.

More knowledge is needed on the work situation and health of managers as they enter or leave managerial positions [16]. Managerial transitions have only been investigated in a few studies. Li et al. [34] found that the level of demands and control increased when entering a managerial position but without influencing their well-being. Li et al. [34] did not, however, include individuals who left the managerial position. On the other hand, West et al. [49] found a deterioration in well-being when leaving a managerial position, but their study did not include individuals entering a managerial position. For a better understanding of managerial transitions, individuals entering or leaving a managerial position need to be compared with individuals remaining as managers and non-managers in the same organization and during the same time period.

### 1.2 Hypotheses based on the favourable conditions and the selection effect explanation

This section describes the results that can be expected based on the two explanations. It starts with the favourable condition explanation and continues with the selection effect explanation.

**Fig. 1:**
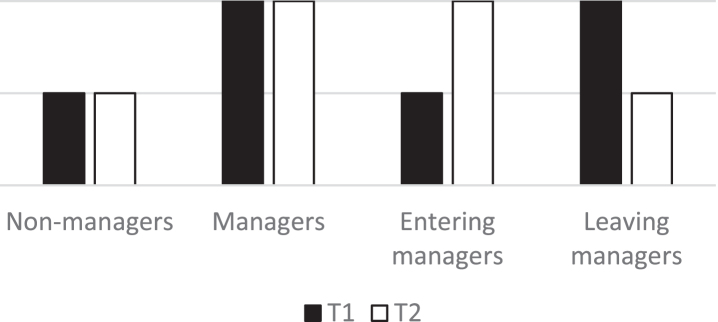
Illustration of how work environment and health should be rated at Time 1 and Time 2 according to the favourable condition explanation.

If the psychosocial work environment is perceived differently because of entering a managerial position, and if health is improved because of an improved work environment, it seems reasonable that non-managers and managers at Time 1 and Time 2 do not change the ratings of their work environment or health between measuring points, as they do not change position (depicted in Fig. 1). In addition, non-managers should generally rate their work environment and health lower than managers. Those who change their position should instead rate their work environment and health differently between the measuring points. Individuals entering a managerial position should at Time 1 rate their work environment and health like non-managers, but their rating should be like managers at Time 2. Conversely, the ratings of individuals leaving a managerial position should be like managers at Time 1 and as non-managers at Time 2.

**Fig. 2:**
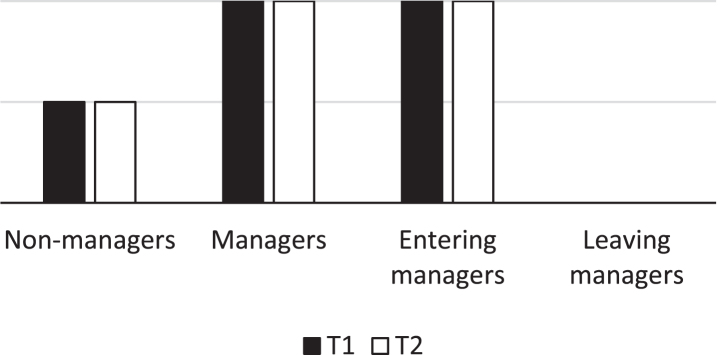
Illustration of how work environment and health should be rated at Time 1 and Time 2 according to the selection effect explanation.

On the other hand, if those who get hired as managers already consider their work environment and health as good, then there should be no differences between those who stay as managers at both Time 1 and Time 2 and those who enter a managerial position. In addition, their rating should be higher than non-managers (depicted in Fig. 2). Those leaving the managerial position constitutes an interesting group. Their ratings of work environment and health could be higher than non-managers, like the other two managerial groups, since they have been recruited to a managerial position. But their ratings could also be lower than the other two managerial groups since they will leave that position, and the work environment or their health could be part of the reasons for it [8, 9, 50].

## 2 Method

### 2.1 Sample and procedure

The material consists of a longitudinal self-reported questionnaire answered at two time points by 1971 employees within seven larger organizations. A questionnaire was sent to 7319 employees in seven organizations in Sweden, and 4210 questionnaires were returned (Time 1), giving a response rate of 58%. In the follow up two years later (Time 2), 2945 responders were still employed and received a second questionnaire. 1971 questionnaires were returned (response rate 67%) and constitute the final longitudinal sample.

The seven organizations consisted of one private production company (*n* = 329), one private care company (*n* = 297), two municipal organizations (*n* = 511, *n* = 130), one public care organization (*n* = 74), and two governmental authorities (*n* = 354, *n* = 276). Before distributing the questionnaire, organizational schemes and lists containing names, positions (e.g., manager/non-manager), age, and gender of employees were collected from the organizations. The responders were coded to determine how they were nested within the organizations. The mean age was 49 years at follow up (*SD* = 10.07), and 66% were women. The majority (51%) had a secondary educational degree, and 39% had a university degree.

### 2.2 Measurements

#### 2.2.1 Psychosocial work environment

To measure demands, control, skill discretion and decision authority, the Swedish Demand Control Questionnaire was used [10, 51]. Demands consist of five items investigating time pressure, role conflicts, and mental workload at work. An example item is: Does your job require you to work very fast?. Skill discretion consists of four items investigating the ability to use and develop skills at work. An example item is: Does your job require creativity?. Decision authority consists of two items investigating the influence on work tasks. An example item is: Do you have the possibility to decide for yourself how to carry out your work?. The response scale ranges from Yes, often (1) to No, never (4). The index was reversed. Control is the combination of skill discretion and decision authority. In this study, Cronbach’s alpha was .80, .60, .79 and .69 for demands, skill discretion, decision authority and control.

To capture the social aspects in the extended demands-control-support model [10], the workplace social capital scale was used [52]. The eight items concern whether people feel respected, valued, and treated as equals at work. An example item is: People feel understood and accepted by each other. The response scale ranges from Fully disagree (1) to Fully agree (5). Cronbach’s alpha was .90.

#### 2.2.2 Indicators of health

Two indicators of health were used to capture the concept from a pathogenic and a salutogenic perspective [26, 27]. Both instruments have been used previously in relation to the demands-control-support model. The first one address health from a pathogenic perspective and regards symptoms of burnout, involving complaints and lack of energy. Symptoms of burnout were measured by the generic part of the Copenhagen Burnout Inventory (CBI) [53]. The scale is intended to answer the question How tired or exhausted are you?, and the response scale is a 5-point Likert scale (1 = Always, 5 = Never/almost never). The range of the scale is 0–100, where the first category (always) is scored 100, and the fifth category (never/almost never) is scored 0. Cronbach’s alpha was .89.

The second indicator of health concerns flow at work. The concept originates from a positive tradition of occupational research and is used here as an indicator of health from a salutogenic perspective [54]. Flow at work was measured by the Work-related flow inventory (WOLF) [55]. The scale consists of 13 items and indicates whether individuals have experienced flow at work during the preceding two weeks, in terms of absorption, intrinsic motivation, and enjoyment. An example item is: When I am working, I think about nothing else. The response scale ranges from Never (1) to Always (5). Cronbach’s alpha in this study was .86.

### 2.3 Statistical analyses

The participants were coded by comparing the organizational schemes collected at Time 1 and Time 2. Those who were managers at both Time 1 and Time 2 were labelled Managers. Those who were non-managers were labelled Non-managers. Those who were non-managers at T1 but managers at T2 were labelled Entering managers, and similarly those who were managers at T1 but non-managers at T2 were labelled as Leaving managers.

Analysis of variance (ANOVA) was performed for Time 1 and Time 2 to compare the means of the four groups on psychosocial work environment and indicators of health. To control for possible differences between the organizations, the analysis was adjusted for organizational affiliation (e.g., in which organization they were employed). Bonferroni *post hoc* test was used. To test the interaction between group belonging and time on work environment and indicators of health, ANOVA with a mixed design was performed, adjusted for organizational affiliation. This analysis was followed by an ANOVA comparing the change in mean (Time 2 minus Time 1) of the investigated variables, adjusted for organizational affiliation. Bonferroni *post hoc* test was used.

To ensure the validity of the results, as ANOVA can be sensitive to different sizes of the groups, multiple linear regression analyses with dummy variables was performed, and Entering managers and Leaving managers were used as the comparison group. The analyses were adjusted for organizational affiliation. The psychosocial work environment and indicators of health of the four groups at Time 1, Time 2, and their change (Time 2 minus Time 1) were also compared using Kruskal-Wallis tests, with complementary Mann-Whitney U-tests (Bonferroni corrected). As the conclusion of the regression analyses and the Kruskal-Wallis tests supported the conclusions of the ANOVAs, these are not reported in the paper. SPSS version 25.0 was used.

## 3 Results

### 3.1 Psychosocial work environment and indicators of health at Time 1

To investigate if perceived psychosocial work environment and indicators of health differed between Non-managers, Managers, Entering managers, and Leaving managers at Time 1, an ANOVA was conducted, adjusted for organizational affiliation.

The result showed significant differences in all investigated variables (see Table 1). *Post hoc* tests showed that most differences were found between Non-managers and Managers. Entering managers rated higher control, skill discretion and flow compared to Non-managers, and lower demands than Managers. Leaving managers rated higher demands, control and skill discretion compared to Non-managers. No differences were found between Managers and Leaving managers.

**Table 1:** ANOVA of variables at Time 1, adjusted for organizational affiliation

	1. Non-managers (*n* = 1722)	2. Managers (*n* = 190)	3. Entering managers (*n* = 31)	4. Leaving managers (*n* = 21)
Variables	*EMM (SE)*	*EMM (SE)*	*EMM (SE)*	*EMM (SE)*	*ANOVA*	*Post hoc*
Demands	13.78 (0.07)	15.14 (0.19)	13.61 (0.45)	15.21 (0.55)	*F*(3;1949) = 16.90, *p*<.001	1 : 2,4 2 : 1,3
Control	18.06 (0.07)	20.23 (0.19)	19.64 (0.44)	19.39 (0.54)	*F*(3;1953) = 43.78, *p*<.001	1 : 2,3,4
Skill discretion	12.23 (0.05)	13.66 (0.12)	13.33 (0.29)	13.07 (0.35)	*F*(3;1932) = 45.13, *p*<.001	1 : 2,3,4
Decision authority	5.84 (0.04)	6.58 (0.11)	6.32 (0.26)	6.33 (0.31)	*F*(3;1948) = 15.18, *p*<.001	1 : 2
Social capital	3.82 (0.02)	4.12 (0.05)	3.89 (0.13)	4.01 (0.15)	*F*(3;1952) = 9.76, *p*<.001	1 : 2
CBI	35.47 (0.50)	29.96 (1.36)	32.69 (3.15)	36.94 (3.83)	*F*(3;1954) = 5.34, *p* = .001	1 : 2
Flow	3.13 (0.02)	3.50 (0.04)	3.43 (0.10)	3.27 (0.12)	*F*(3;1948) = 25.59, *p*<.001	1 : 2,3

### 3.2 Psychosocial work environment and indicators of health at Time 2

To investigate if psychosocial work environment and indicators of health differed between the four groups at Time 2, where Entering managers had a managerial position and Leaving managers no longer were managers, an ANOVA was conducted, adjusted for organizational affiliation.

The result showed significant differences in all variables (see Table 2). *Post hoc* tests showed differences between Non-managers and Managers in all variables. Differences were also found between Non-managers and Entering managers, and between Non-managers and Leaving managers concerning control, skill discretion, decision authority and flow at work. Differences in flow at work were found between Non-managers and Entering managers. No differences were found between Managers, Entering managers, or Leaving managers.

**Table 2:** ANOVA of variables at Time 2, adjusted for organizational affiliation

	1.Non-managers (*n* = 1710)	2.Managers (*n* = 189)	3.Entering managers (*n* = 31)	4.Leaving managers (*n* = 21)
Variables	*EMM (SE)*	*EMM (SE)*	*EMM (SE)*	*EMM (SE)*	*ANOVA*	*Post hoc*
Demands	13.63 (0.08)	14.93 (0.21)	14.61 (0.48)	13.78 (0.58)	*F*(3;1940) = 13.08, *p*<.001	1 : 2
Control	18.02 (0.07)	20.28 (0.19)	20.35 (0.45)	19.81 (0.54)	*F*(3;1941) = 51.10, *p*<.001	1 : 2,3,4
Skill discretion	12.11 (0.05)	13.50 (0.13)	13.45 (0.30)	13.02 (0.37)	*F*(3;1921) = 40.70, *p*<.001	1 : 2,3,4
Decision authority	5.91 (0.04)	6.77 (0.11)	6.89 (0.25)	6.79 (0.31)	*F*(3;1930) = 25.23, *p*<.001	1 : 2,3,4
Social capital	3.83 (0.02)	4.02 (0.06)	3.99 (0.13)	3.97 (0.16)	*F*(3;1934) = 4.32, *p* = .005	1 : 2
CBI	35.47 (0.52)	30.55 (1.41)	34.04 (3.27)	31.64 (3.96)	*F*(3;1939) = 3.98, *p* = .008	1 : 2
Flow	3.09 (0.02)	3.42 (0.04)	3.48 (0.10)	3.32 (0.12)	*F*(3;1933) = 23.49, *p*<.001	1 : 2,3

### 3.3 Changes in psychosocial work environment and indicators of health

To investigate if psychosocial work environment and indicators of health of the four groups had changed between the two measuring points, an ANOVA with a mixed design was conducted, adjusted for organizational affiliation. The result showed a significant interaction effect between group and time on demands (*F*(3;1928) = 4.04, *p* = .007), and an almost significant interaction effect (*F*(3;3;1917) = 2.52, *p* = .056) between group and time on decision authority.

To clarify the result of the mixed design ANOVA, an additional ANOVA was performed to investigate which group had changed the most using delta variables (delta = Time 2 minus Time 1), adjusted for organizational affiliation. *Post hoc* tests showed that Entering managers had significantly increased their demands compared to the other three groups, and almost significantly (*p* = .056) increased their decision authority compared to Non-managers (see Table 3).

**Table 3:** ANOVA of the change between Time 1 and Time 2, adjusted for organizational affiliation

	1.Non-managers (*n* = 1703)	2.Managers (*n* = 189)	3.Entering managers (*n* = 31)	4.Leaving managers (*n* = 21)
Variables	*EMM (SE)*	*EMM (SE)*	*EMM (SE)*	*EMM (SE)*	*ANOVA*	*Post hoc*
*Δ*Demands	–0.15 (0.07)	–0.22 (0.20)	1.00 (0.45)	–1.43 (0.55)	*F*(3;1928) = 4.04, *p* = .007	3 : 1,2,4
*Δ*Control	–0.04 (0.06)	0.08 (0.18)	0.73 (0.40)	0.42 (0.49)	*F*(3;1933) = 1.56, *p* = .197
*Δ*Skill discretion	–0.12 (0.05)	–0.13 (0.12)	0.15 (0.28)	–0.05 (0.34)	*F*(3;1895) = 0.31, *p* = .820
*Δ*Decision authority	0.07 (0.04)	0.20 (0.10)	0.59 (0.24)	0.46 (0.29)	*F*(3;1917) = 2.52, *p* = .056	3 : 1
*Δ*Social capital	0.01 (0.02)	–0.10 (0.06)	0.10 (0.14)	–0.04 (0.17)	*F*(3;1926) = 1.18, *p* = .315
*Δ*CBI	0.02 (0.44)	0.71 (1.18)	1.29 (2.75)	–5.28 (3.34)	*F*(3;1933) = 1.04, *p* = .374
*Δ*Flow	–0.04 (0.01)	–0.08 (0.04)	0.05 (0.08)	0.05 (0.10)	*F*(3;1921) = 1.10, *p* = .349

### 3.4 The combination of demands and control at Time 1 and Time 2

The distribution of the four groups in the four work situations described in the demands-control model was also investigated (see Table 4). The result showed that Non-managers were generally equally distributed across all four work situations, whereas Managers were concentrated to the two situations high in control. The distribution of Non-managers and Managers across the work situations did not change much between Time 1 and Time 2. However, the distribution of those who changed their position did change between Time 1 and Time 2. The proportion of Entering managers who rated their work situation as low in demands, low in control decreased between Time 1 and Time 2, while those who rated their work situation as high in demands, high in control increased. For Leaving managers, on the other hand, the proportion who rated their work situation as high in demands, high in control decreased, while the proportion who rated their work situation as low in demands, high in control increased.

**Table 4:** Number (percent) of non-managers, managers, entering managers, leaving managers at Time 1 and Time 2 distributed over the four combinations of demands and control

	Non-managers		Managers		Entering managers		Leaving managers	
	*T1*	*T2*	*T1*	*T2*	*T1*	*T2*	*T1*	*T2*
Combinations	*n (%)*	*n (%)*	*n (%)*	*n (%)*	*n (%)*	*N (%)*	*n (%)*	*n (%)*
Low demands, low control	326 (19)	351 (21)	11 (6)	13 (5)	7 (23)	2 (7)	2 (10)	1 (5)
Low demands, high control	472 (28)	474 (28)	64 (34)	75 (40)	13 (42)	11 (36)	6 (29)	11 (52)
High demands, high control	540 (31)	547 (28)	106 (56)	90 (48)	7 (23)	16 (52)	12 (57)	8 (38)
High demands, low control	380 (22)	336 (20)	8 (4)	11 (6)	4 (13)	2 (7)	1 (5)	1 (5)
Total	1718 (100)	1708 (100)	189 (100)	189 (100)	31 (100)	31 (100)	21 (100)	21 (100)

## 4 Discussion

The purpose of this study was to increase the understanding of managers’ psychosocial work environment and health by testing the two explanations on individuals entering or leaving a managerial position. The results show that managers rate higher levels of demands, resources, and health than non-managers, which is in line with previous research [11, 12, 28–30, 32]. But the present research went further by testing two different explanations for managers’ ratings of their work conditions and health: favourable conditions of the managerial position and the selection effect of those hired as managers.

The result shows that entering a managerial position increases the level of demands and decision authority. Even before entering the managerial position, these individuals rated higher levels of control, skill discretion and flow compared to non-managers. Those leaving a managerial position decreased the level of demands, while available resources remained in terms of control, skill discretion and decision authority. Thus, employees entering a managerial position generally seem to transition from a low demands, high control situation to a high demands, high control situation (an active job situation according to the model [10]). Employees leaving a managerial position, on the other hand, seem to transition from a high demands, high control situation to a low demands, high control situation (an ideal/low strain job situation according to the model [10]).

These results, therefore, partially support both investigated hypotheses. Entering a managerial position does have consequences for the perceived psychosocial work environment, especially in terms of increased demands. But selection effects also exist as these employees rated a more favourable psychosocial work environment and better health before entering the position. It seems that individuals who enter a managerial position have a more positive view of their organization and workplace. It may very well be their access to resources that allow them to be committed, driven and therefore considered a high potential [36, 45, 46]. The perceived demands of those that left the managerial position, on the other hand, decreased. Interestingly though, despite changes in the level of demands of managers entering or leaving, their health did not significantly increase or decrease. Previous research has found that the level of burnout increased in job changers initially but decreased over time [56], likely because a new position means new role expectations and new work tasks etc. [57]. Therefore, the results are in line with Li et al. [34], who found that the managerial position had little effect on health, but in contrast to findings showing decreased health in demoted managers [49]. West et al. [49] suggest that the reason for the impaired well-being of the demoted managers in their study was the loss of the status associated with being a manager. The managers in the present study remained in the organization, and their new role may not have weakened their social status, e.g., by retaining unofficial authority.

For those who left the managerial position, the job resources remained high, in line with previous research showing that resources at work are stable over time and do not change because of career transitions [57, 58]. The fact that the resources did not decrease when the managers left their managerial position further strengthens the suggestion that they transitioned into highly qualified positions and retained authority and status at work, even if it was not a managerial one.

It is important to remember that organizations may differ in the demands and resources available in different jobs and positions [59–61]. The analyses were adjusted for organizational affiliation, but potential differences within the organization have not been addressed. Similar differences may be found at different managerial levels [11, 15, 35, 62]. In this study, all were first-line managers, and no one entered or left a middle or higher managerial level. An interesting venue in future research would be to analyse potential differences between different jobs, organizations, or between different managerial levels. Some studies suggest that lower managerial levels are more demanding and stressful than higher managerial levels [11, 15, 35, 62]. Since most in higher managerial levels previously have had a lower position, it is possible that being promoted does not increase the perceived demands, only the available resources.

### 4.1 Limitations

In addition to the limitations mentioned above, the study has other limitations. The uneven distribution between the investigated groups is, of course, a limitation. However, several different statistical procedures have been used to minimize potential bias, and they have all shown similar results.

A further limitation concerns the investigated variables. The demands-control-support model [10] is one of the most well-used models to investigate psychosocial work environment and health [17–20] and was therefore used here as well. Nevertheless, there may be different types of demands and other types of resources apart from the ones investigated in the present research [63]. The demands scale was a generic measure, but it would be useful to investigate how different types of demands are affected by job changes. Differentiating between time-based demands, emotional demands, etc., would further help organizations to provide relevant support.

Another limitation is that information was only available regarding if employees had changed position, but not when they had done so or the specific reason for changing position. That information may be important as long-term changes in health can take time. On the other hand, flow at work was also examined, which is considered to be more dynamic [54, 55].

## 5 Conclusions and implications

This study shows that the work situation does change when entering or leaving a managerial position. But the study also shows that individuals employed as managers even before entering the managerial position had high levels of resources, which was maintained after leaving that position. The study, therefore, contributes to the understanding of the differences between managers and non-managers by showing that it is a combination of available resources in the position and resources that are brought into the position. The favourable conditions and the selection effect explanation are both valid and should be combined to understand what happens when someone enters or leaves a managerial position. However, only a few resources (of the ones investigated in this paper) were influenced by position, which emphasizes the organizations’ opportunities to build resources for employees and future managers. A thorough and well-built talent management program may highlight the organizations’ willingness to invest in their employees and consider them as future managers. Organizations’ can and should therefore increase the resources of their employees and develop supportive strategies for newly hired managers. Although increased demands can be expected when getting promoted [57], it is hardly beneficial with overwhelming demands [64]. Developing supportive strategies for managers may ease their stressful work situation and lessen their perception of being required to handle problems on their own that should be managed with others [45]. Strategies aimed at building resources and helping managers may result in more employees considering managerial assignments as a developmental opportunity, which could mitigate the current problems of recruiting and retaining managers [8, 9].
